# Reproductive Outcome of the Patients with Uterus Unicornis: Five Year Experience in a Tertiary Center

**DOI:** 10.15388/Amed.2022.29.2.14

**Published:** 2022-06-29

**Authors:** Sule Atalay Mert, Berna Dilbaz, Ece Sevin Cukurova, Caner Kose, Yaprak Engin Ustun

**Affiliations:** University of Health Sciences, Etlik Zubeyde Hanim Women’s Health, Training and Research Hospital, Department of Reproductive Endocrinology and IVF, Ankara, Turkey; University of Health Sciences, Etlik Zubeyde Hanim Women’s Health, Training and Research Hospital, Department of Reproductive Endocrinology and IVF, Ankara, Turkey; University of Health Sciences, Etlik Zubeyde Hanim Women’s Health, Training and Research Hospital, Department of Reproductive Endocrinology and IVF, Ankara, Turkey; University of Health Sciences, Etlik Zubeyde Hanim Women’s Health, Training and Research Hospital, Department of Reproductive Endocrinology and IVF, Ankara, Turkey; University of Health Sciences, Etlik Zubeyde Hanim Women’s Health, Training and Research Hospital, Department of Reproductive Endocrinology and IVF, Ankara, Turkey

**Keywords:** Müllerian malformations, laparoscopic surgery, hysteroscopy, rudimentary horn, uterus unicornis

## Abstract

**Aim:**

The aim is to evaluate the pregnancy outcomes of patients with uterus unicornis for 5-year experience in a tertiary center.

**Material and Method:**

Twenty patients with uterus unicornis who were diagnosed with hysterosalpingography and/or 3D TVUSG between 2017 and 2021 and then confirmed with laparoscopy and simultaneous hysteroscopy were recruited into this retrospective study. The reproductive outcome and obstetric complications of the patients were followed up for two years postoperative period.

**Results:**

Overall 20 patients who fulfilled the inclusion criterion were recruited for the study. The mean age was 28.65±5.03 years. Thirteen patients (65%) had primary infertility while the remaining seven had secondary infertility with two of them having a previous ectopic pregnancy. Rudimentary communicating uterine horn was observed in 7 (35%) of the patients. The horns were excised during laparoscopy. Overall, 14 (70%) pregnancies were achieved during the 2 years follow-up period. Twelve (85.7%) patients had a live birth (71.4% term delivery, 21.4% preterm delivery), and two (15.3%) had a first-trimester spontaneous abortion. Six (50%) of 12 patients who had a live birth received tocolytics for preterm labor.

**Conclusion:**

Unicorn uterus is a rare anomaly diagnosed mostly during infertility work-up and might be related to the poor obstetric outcome, but early diagnosis is important for successful pregnancy results for preterm delivery especially in the secondary infertile group. In addition, rudimentary horn excision is recommended due to the high risk of obstetric complications.

## Santrauka.

**Tikslas:**

Šio tyrimo tikslas yra įvertinti nėštumo baigtį pacienčių, turinčių vienaragę gimdą, apibendrinant 5 metų darbo patirtį trečiojo lygmens sveikatos priežiūros centre.

**Medžiagos ir metodai:**

Nuo 2017 iki 2021 metų vienaragė gimda buvo diagnozuota 20 pacienčių, pasitelkus histerosalpingografiją ir / arba trimatę transvagınalınę ultrasonografıją. Šie rezultatai tuomet buvo patvirtinti atlikus laparoskopiją ir histeroskopiją. Visos pacientės buvo pakviestos dalyvauti šiame retrospektyviame tyrime. Dėl poveikio vaisingumui ir akušerinių komplikacijų jos buvo stebimos pooperaciniu laikotarpiu iki dvejų metų.

**Rezultatai:**

Tyrime dalyvavo 20 pacienčių, atitikusių įtraukimo kriterijų. Vidutinis dalyvių amžius 28,65±5,03 metai. Trylikai pacienčių (65 %) buvo pradinis nevaisingumas, o kitos septynios pacientės patyrė antrinį nevaisingumą; dvi iš jų buvo patyrusios ektopinį nėštumą. Rudimentinis susisiekiantis gimdos ragas nustatytas 7 (35 %) pacientėms, jis pašalintas, pasitelkus laparoskopiją. İš viso per dvejų metų stebėjimo (tolesnio stebėjimo) laikotarpį buvo pasiekta 14 (70 %) nėštumų. Dvylika iš 14 pacienčių (85,7 %) pagimdė gyvus vaikus (71,4 % gimdė laiku, 21,4 % priešlaikinis gimdymas), o dviem pacientėms įvyko pirmojo semestro spontaninis persileidimas (15,3 %). Šešioms (50 %) iš 12 pacienčių, pagimdžiusių gyvus vaikus, buvo skiriami tokolitai priešlaikinės gimdymo veiklos laikotarpiu.

**Išvada:**

Vienaragė gimda yra reta anomalija, ji įprastai nustatoma tiriant nevaisingumą ir gali būti susijusi su nepalankiomis akušerinėmis baigtimis, todėl anksti diagnozuoti šią anomaliją yra svarbu, kad būtų pasiekta sėkmingų nėštumo rezultatų, visų pirma priešlaikinio gimdymo atveju ir ypač esant antrojo lygmens nevaisingumui. Be to, didelio akušerinių komplikacijų pavojui išvengti rekomenduojama pašalinti rudimentinį ragą.

## Introduction

The prevalence of Müllerian duct anomalies (MDAs) is estimated to be 4.3% in the general population [1], while the incidence is 3.5% in infertile women and 13% in cases with recurrent pregnancy loss [2]. The patients with uterus unicornis constitute 5–20% of congenital uterine anomalies [3, 4]. The presence of a rudimentary horn accompanying the uterus unicornis is a rare Mullerian anomaly with a rate of 1/100,000 [1, 5]. In the r-ASRM classification system, the uterus unicornis is evaluated according to the presence of a rudimentary horn (cavity-related or unrelated). Urinary system anomalies might accompany this anomaly, which constitutes 1–3% of congenital Müllerian anomalies [6] .

In women with a uterus unicornis, approximately 1 out of every 76,000 pregnancies is accompanied by a rudimentary horn, while 83% of these cases were reported to be ectopic pregnancies [7, 8]. Other reported obstetric complications related to the uterus unicornis are rupture of the uterine horn, cervical insufficiency, pregnancy-induced hypertension, intrauterine growth retardation (IUGR), and postpartum hemorrhage [7]. Early diagnosis of Müllerian anomalies gains importance in terms of being careful about possible obstetric complications. Although patients with uterus unicornis are generally associated with poor obstetric history and infertility, it is also known that they have term and live births [9].

While pregnancy complications such as ectopic pregnancies and rudimentary horn pregnancies are mainly evaluated with case reports on patients with uterus unicornis, in the presented study we aimed to evaluate the pregnancy outcomes of patients with uterus unicornis for 5 years in a tertiary center.

## Material and Method

This retrospective study was conducted at the Health Sciences University, Etlik Zubeyde Hanim Women’s Health Research and Training Hospital, Department of Reproductive Endocrinology, and Infertility Clinic. The infertile patients who were diagnosed with uterus unicornis by hysterosalpingography (HSG) and/or 3D TVUSG between January 2017 and January 2021, and whose diagnoses were confirmed by laparoscopy and hysteroscopy and followed-up for 2 years were recruited. The data of the patients were abstracted from the electronic medical records that included the special forms developed for patients with uterine anomalies. In all patients, 2-dimensional ultrasonography was also performed for ruling out the presence of accompanying urinary tract anomalies.

This retrospective study involving human participants was in accordance with the ethical standards of the institutional and national research committee and with the 1964 Helsinki Declaration and its later amendments or comparable ethical standards. The study protocol was approved by the institutional review board of Health Sciences University, Etlik Zubeyde Hanim Women’s Health Research and Training Hospital (Date: 21.04.2022, No:05/19). As a hospital policy, before each procedure, written informed consent was obtained from each patient that gave permission to use the medical data anonymously for future studies.

Sociodemographic characteristics of the patients (age, gravida, parity, type of infertility, duration of infertility), laparoscopic and hysteroscopic findings, and operative procedure if performed excision of the rudimentary horn were recorded. The obstetric outcome time to achieve pregnancy, obstetric complications, type of delivery, and the birth weight of the newborn were recorded in the patient follow-up forms.

Patients of the reproductive age (18–40 years of age) who were diagnosed with uterus unicornis were included in the study. Patients with additional causes of infertility, such as premature ovarian failure, male factor, ovulatory dysfunction, tubal factor, endometriosis, coital problems, or who had an accompanying uterine factor (leiomyoma, endometrial polyps) or vaginal anomaly accompanying uterine anomaly were excluded from the study.

## Pre-operative Procedures

The diagnosis of the uterus unicornis was performed by hysterosalpingography (HSG) and/or 3D TVUSG [Fig.1 (HSG) and Fig 2 (3D TVUSG)]. All patients were evaluated by the same team using 3D TVUSG (Samsung H570A, 5–6 MHz endovaginal probe manufactured by Samsung Medison Co., LTD in Seoul, Korea) and the urinary system was evaluated by 2D ultrasonography.

## Operative Procedures

All the procedures were performed under general anesthesia in the early follicular phase of the menstrual cycle. No endometrial preparation was given before the procedure. A laparoscopy with a simultaneous hysteroscopy was performed. During laparoscopic surgery, tubal patency was evaluated with a methylene blue dye test during laparoscopy. During hysteroscopy, the panoramic view was assessed, and a very narrow cavity, with unilateral cornual area and ostium, was taken as the confirmation of the diagnosis. In cases when a rudimentary horn was diagnosed, the peritoneum was dissected and the bladder was freed from the uterus (Fig. 3). Then, the rudimentary horn was excised from the communicating area with the preservation of the uterus unicornis. The excision was performed using Covidien Liga Sure (Medtronic Co., Davis & Geck Caribe Limited, Dublin, İreland). After the bleeding was controlled, the rudimentary horn was removed completely outside the abdomen with an Endobag (Medtronic Co., Davis & Geck Caribe Limited, Dublin, İreland), and sent to pathology. No early complications were observed and the procedure was terminated. All the patients were followed up until May 2022 for evaluation of the reproductive outcome. The ART (Assisted reproductive techniques) method used to achieve pregnancy was recorded. The reproductive outcome was given as abortion, preterm or term delivery, stillbirth, and ectopic pregnancy.

**Fig. 1a.**
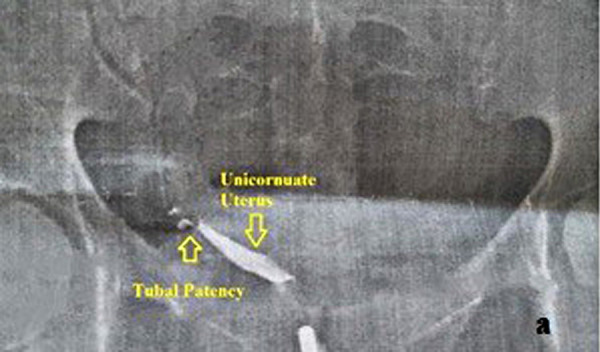
Hysterosalpingographic image of the unicornuate uterus

**Fig. 1b.**
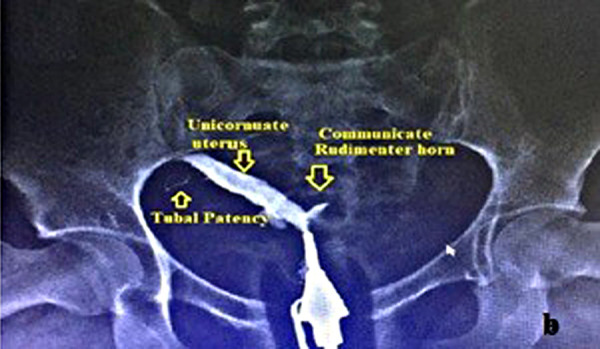
Hysterosalpingographic image of the unicornuate uterus with communicate rudimentary horn

**Fig. 2.**
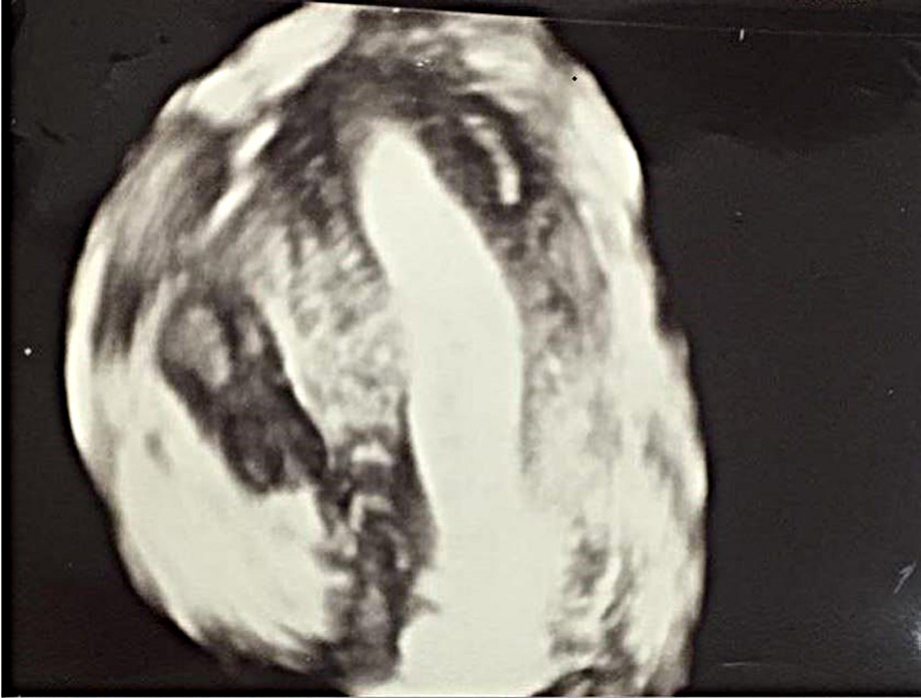
3D TVUSG image of the unicornuate uterus

**Fig. 3.**
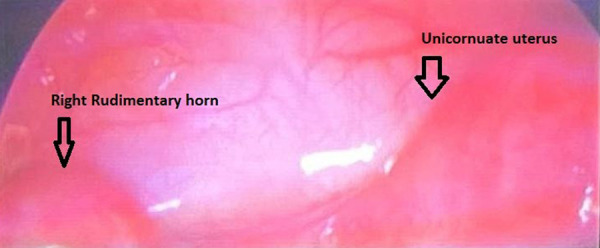
Figure of the rudimentary horn and unicornuate uterus at laparoscopic surgery

## Statistical Method

Statistical analysis was performed using IBM SPSS 26.0 software (IBM Corp. Released 2019. IBM SPSS Statistics for Windows, Version 26.0. Armonk, NY: IBM Corp). Frequency values were expressed as percentages. Demographic variables, medical characteristics, pregnancy rates, and the treatment protocol used for achieving pregnancy and pregnancy outcome were obtained from the hospital records and the findings were analyzed.

## Results

Overall 20 patients who fulfilled the inclusion criterion and were diagnosed with uterus unicornis were recruited into the study. The mean age of the patients was 28.65±5.03 (range=22–39) years. Thirteen patients (65%) had primary infertility, while the remaining seven patients (35%) had secondary infertility. Among the patients with secondary infertility, two had a history of ectopic pregnancy of which one had a rudimentary horn, and only one patient had a history of recurrent pregnancy loss. Demographic characteristics were detailed in Table 1. Only one of the patients had ipsilateral renal agenesis. No urinary or skeletal anomalies were detected in the remaining 19 patients.

**Table 1.** Demographic Characteristics of the Patients with Uterus Unicornis

	Min.	Max.	Mean± Std. Dev.
Age	22.00	39.00	28.65±5.03
Gravida	.00	7.00	1.41±1.69
Parity	.00	2.00	.82±.80
Extrauterine pregnancy	.00	1.00	.11±.33
Number of the Spontaneous Abortion	.00	5.00	.29±1.21
Duration of Marriage (years)	1.00	8.00	3.88±1.61
Duration of Infertility (years)	1.00	7.00	2.52±1.90
Newborn weight (grams) (N=10)	1950	373.00	2823.75±568.00

Min: Minumum, Max: Maximum, Std. Dev.: Standart Deviation

**Figure 4.**
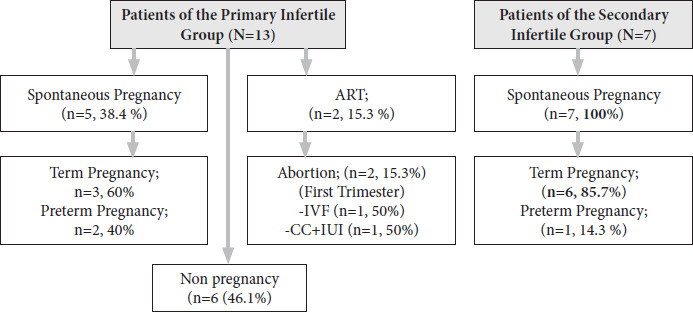
Distrubition of the Pregnancy Outcome Of the Patients with Uterus Unicornis (n=20)

Fourteen patients had pregnancies (70%) after the diagnosis of uterus unicornis. The live birth rate was 85.7%. All pregnancies of 7 patients in the secondary infertile group resulted in live birth (100%), one of them was preterm delivery. In the primary group 3 of the live births were term deliveries while 20% had preterm delivery (Fig. 4). Six (50%) of 12 patients who had a live birth received tocolytics for preterm labor. The mean weight of the newborns was 2823.75±568.70 (range=1950–3730 g) and the mean pregnancy duration was 32.84 ±8.94 weeks (range=11–39 weeks). Two of the women treated for preterm labor reached 34 and 30 weeks of pregnancy.

A rudimentary horn was observed in 7 (35%) of the patients. All of them had a communicating rudimentary horn that was excised during laparoscopy. No obstetric complications such as uterine rupture or ectopic pregnancy were observed after laparoscopic surgery for the rudimentary horn.

## Discussion

Müllerian abnormalities are present in 1.7/1000 fertile women, and a uterus unicornis is observed in infertile 4/1000 women. Their pregnancy results and obstetrics complications are very different. In the presented study we found succesful pregnancy results in the secondary infertile group.

**ART:** Assisted reproductive techniques, **IVF:** Invitro fertilisation, **CC+IUI:** Clomiphene citrate+ intrauterine insemination

For an accurate diagnosis, pelvic examination, urinary tract evaluation, HSG (Hysterosalpingography), MRI (Magnetic Resonance Imaging), and 3D TRUS (Trans Rectal USG) is required in virgin patients. As in our cases, due to the high sensitivity of 3D TVUSG after HSG in the differential diagnosis of uterine malformations. 3D TVUSG is now replacing MRI in most settings. There is a high concordance between 3D TVUSG and MRI results in classifying different anomalies based on the ESHRE–ESGE consensus [10]. Congenital anomalies of the Müllerian system are usually diagnosed during infertility workup. These imaging techniques should also be used for the detection of urinary system anomalies that might accompany the uterus unicornis [11, 12]. In the presented study 3D or 2D TVUSG was used to evaluate urinary system anomalies and one patient was diagnosed to have ipsilateral renal agenesis similar to the reported cases in the literature [13].

Reichman et al. presented a review that covered twenty studies from 1953 to 2006 and reported 7% ectopic pregnancy, 34% first- and second-trimester abortion, 20.1% preterm delivery, 10.5% intrauterine fetal demise, and 49.9% live birth in 290 women with uterus unicornis [14]. In the presented study total of the live birth rate was 85.7% (12 of 14), while it was 100% (7 of 7) in the patients of the secondary infertile group. The term pregnancy rate was 71.4% (10 of 14), the preterm delivery rate was 21.4% (3 of 14), and first-trimester abortion rate was 15.3% (2 of 14). We did not see ectopic pregnancy after the surgery for the unicorn uterus, but before the diagnosis two of the patients had ectopic pregnancy history.

When the pregnancy results of assisted reproductive techniques in women with a uterus unicornis were evaluated, the pregnancy rate and newborn weights were found to be lower in patients when compared to the women with normal uterine cavities [15]. In another study, it was shown that 34 patients with a uterus unicornis who underwent an IVF-ET cycle had a high chance of conceiving and giving birth to a healthy newborn, but the risk of obstetric complications was high [16]. In the presented study all live births were achieved through natural fecundation (spontaneous pregnancy), and term pregnancy was achieved in 100% in the secondary infertile group. Newborn weights (2823.75±568.70 (range=1950–3730 g)) were found to be the similar to term deliveries, too.

The prevalence of uterus unicornis was reported to be higher in the infertile population when compared with the general women population (0.5% vs 0.1%) [17]. The relation between uterus unicornis and infertility has been investigated and the incidence of subfertility was reported to be 23.7% in 3181 women with uterus unicornis [18]. Chen et al. reported a lower clinical pregnancy rate in 342 women with the uterus unicornis in comparison to the control group with a lower implantation rate [19]. In the presented study we evaluated only infertile patients with the uterus unicornis retrospectively.

Uterine volume, cervical incompetency, altered placental blood flow, and cervical insufficiency are other factors that are speculated to be related to the higher incidence of subfertility in women with uterus unicornis [20]. Endometriosis is also encountered in women with a uterus unicornis, especially in presence of a noncommunicating functional rudimentary horn [21]. The common complaints in the patients with Mülerian anomalies are cited as dysmenorrhea, severe pelvic pain, infertility, and acute abdominal pain due to the complications related to the functional noncommunicating rudimentary horn [5]. None of the patients in the presented series had pain symptoms or endometriosis detected during laparoscopy, and all of the patients had infertility (primary and secondary).

A uterus unicornis with a rudimentary horn is a rare congenital uterine anomaly that results from the developmental arrest of the two Müllerian ducts with the simultaneous incomplete fusion of the opposite side [22]. Although both uterus unicornis and rudimentary horn are related to poor reproductive outcome a remarkable majority of the published data about the reproductive outcome and obstetric complications are presented as case reports [23–25]. In the presented study we made laparoscopically surgery for rudimentary horns to remove them and we did not see obstetrics complications such as ectopic pregnancy and rudimentary horn rupture after the surgery.

Pre-pregnancy evaluation of the risks for rudimentary horn pregnancy is important. The rudimentary horn is removed either by laparoscopy or laparotomy in order to avoid obstetric complications that range from the rupture of the rudimentary horn during pregnancy to placentation anomalies such as placenta percreata [26]. In addition, as seen in the literature, obstetric complications such as uterine rupture [27], cornual pregnancy, and ectopic pregnancy [28], are not rare in the presence of a rudimentary horn. In our series, only three of the patients had a preterm delivery, and six (50%) of 12 patients who had a live birth received tocolytics for preterm labor. Therefore, early diagnosis is important and excision of the rudimentary horn before pregnancy is strongly recommended.

The limitation of the study is in that it was conducted retrospectively with the recruitment of patients with the uterus unicornis in a 5 years period and the reproductive and obstetric outcome was analyzed. Further studies with a larger number of recruited patients and a comparison of the results with the control group are required.

## Conclusion

Although uterus unicornis is a rare anomaly, a higher incidence is encountered in infertile patients. Although the pregnancy outcomes of patients with uterus unicornis are generally poor, it should not be ignored that they have successful pregnancy outcomes (85.7%), especially patients of the secondary infertile group (100%). Early diagnosis and management are important in the avoidance and early recognition of obstetric complications.
